# Diagnostic Accuracy of Highest-Grade or Predominant Histological Differentiation of T1 Colorectal Cancer in Predicting Lymph Node Metastasis: A Systematic Review and Meta-Analysis

**DOI:** 10.14309/ctg.0000000000000673

**Published:** 2024-01-02

**Authors:** Jun Watanabe, Katsuro Ichimasa, Yuki Kataoka, Shoko Miyahara, Atsushi Miki, Khay Guan Yeoh, Shigeo Kawai, Fernando Martínez de Juan, Isidro Machado, Kazuhiko Kotani, Naohiro Sata

**Affiliations:** 1 Department of Surgery, Division of Gastroenterological, General and Transplant Surgery, Jichi Medical University, Shimotsuke, Tochigi, Japan;; 2 Division of Community and Family Medicine, Jichi Medical University, Shimotsuke-City, Tochigi, Japan;; 3 Digestive Disease Center, Showa University, Northern Yokohama Hospital, Tsuzuki-ku, Yokohama, Japan;; 4 Department of Medicine, National University of Singapore, Singapore;; 5 Department of Internal Medicine, Kyoto Min-iren Asukai Hospital, Sakyo-ku, Kyoto, Japan;; 6 Scientific Research WorkS Peer Support Group (SRWS-PSG), Osaka, Japan;; 7 Section of Clinical Epidemiology, Department of Community Medicine, Kyoto University Graduate School of Medicine, Sakyo-ku, Kyoto, Japan;; 8 Department of Healthcare Epidemiology, Kyoto University Graduate School of Medicine/Public Health, Sakyo-ku, Kyoto, Japan;; 9 Department of Medicine, Division of Gastroenterology, Jichi Medical University, Shimotsuke, Tochigi, Japan;; 10 Department of Gastroenterology and Hepatology, National University Hospital, Singapore;; 11 Department of Diagnostic Pathology, Tochigi Medical Center Shimotsuga, Tochigi-City, Tochigi, Japan;; 12 Department of Gastroenterology and Endoscopy Unit, Instituto Valenciano de Oncología, Valencia, Spain;; 13 Endoscopy Unit, Hospital Quiron Salud, Valencia, Spain;; 14 Medicine, Universidad Cardenal Herrrera-CEU, CEU Universities, Valencia, Spain;; 15 Pathology Department, Instituto Valenciano de Oncología, Patologika Laboratory Hospital Quiron Salud and Pathology Department University of Valencia, Valencia, Spain;; 16 CIBERONC, Madrid, Spain.

**Keywords:** colorectal neoplasms, histology, lymphatic metastasis, meta-analysis, systematic review

## Abstract

**INTRODUCTION:**

Treatment guidelines for colorectal cancer (CRC) suggest 2 classifications for histological differentiation—highest grade and predominant. However, the optimal predictor of lymph node metastasis (LNM) in T1 CRC remains unknown. This systematic review aimed to evaluate the impact of the use of highest-grade or predominant differentiation on LNM determination in T1 CRC.

**METHODS:**

The study protocol is registered in the International Prospective Register of Systematic Reviews (PROSPERO, registration number: CRD42023416971) and was published in OSF (https://osf.io/TMAUN/) on April 13, 2023. We searched 5 electronic databases for studies assessing the diagnostic accuracy of highest-grade or predominant differentiation to determine LNM in T1 CRC. The outcomes were sensitivity and specificity. We simulated 100 cases with T1 CRC, with an LNM incidence of 11.2%, to calculate the differences in false positives and negatives between the highest-grade and predominant differentiations using a bootstrap method.

**RESULTS:**

In 42 studies involving 41,290 patients, the differentiation classification had a pooled sensitivity of 0.18 (95% confidence interval [CI] 0.13–0.24) and 0.06 (95% CI 0.04–0.09) (*P* < 0.0001) and specificity of 0.95 (95% CI 0.93–0.96) and 0.98 (95% CI 0.97–0.99) (*P* < 0.0001) for the highest-grade and predominant differentiations, respectively. In the simulation, the differences in false positives and negatives between the highest-grade and predominant differentiations were 3.0% (range 1.6–4.4) and −1.3% (range −2.0 to −0.7), respectively.

**DISCUSSION:**

Highest-grade differentiation may reduce the risk of misclassifying cases with LNM as negative, whereas predominant differentiation may prevent unnecessary surgeries. Further studies should examine differentiation classification using other predictive factors.

## INTRODUCTION

Colorectal cancer (CRC) is the third most common cancer worldwide and the second leading cause of cancer-related deaths (1,2). The current Japanese CRC treatment guidelines identify poor differentiation (PD), submucosal invasion of >1,000 μm, lymphovascular invasion (LVI), and tumor budding grade (BD) 2 or 3 as risk factors of lymph node metastasis (LNM) in patients with CRC (3). Most guidelines and pathology reports, including those in the United States, Europe, and Asia, consider tumor differentiation as a risk factor of LNM in T1 CRC (3–7). Surgical resection with lymph node dissection is recommended for poorly differentiated adenocarcinoma, signet ring cell carcinoma, or mucinous carcinoma identified by histological examination (3–7). Because surgical resection is linked to high morbidity, mortality, and costs and reduced quality of life, compared with endoscopic resection, selecting the appropriate treatment modality is crucial (3–7).

There are 2 classifications of histological differentiation in CRC treatment guidelines: highest grade and predominant (3,4,6). The World Health Organization and International Collaboration on Cancer Reporting classify differentiation according to the highest grade present (6,8). However, the Japanese guidelines and College of American Pathologists Cancer Protocols classify differentiation according to the predominant grade (3,9). The approach to histological differentiation classification varies globally, with some institutions in Europe, South America, and Asia opting for the predominant differentiation classification, establishing that the concept is far from uniform across countries and institutions (10–16). For instance, if a resected tumor is composed of 95% well-differentiated and 5% poorly differentiated tissue, diagnoses of well-differentiated and poorly differentiated tumors would signify using predominant and highest-grade differentiation, respectively.

However, whether predominant or highest-grade differentiation would be a better predictor of LNM in T1 CRC remains to be clarified (17,18). The main indicators of diagnostic accuracy for LNM are sensitivity and specificity (19). While highest-grade differentiation identifies all cases with PD within the tumor through comprehensive examination of serial paraffin-embedded tissue sections, predominant differentiation is based on most of the tumor. Therefore, highest-grade differentiation tends to overestimate malignancy (18), which may elevate the risk estimation of LNM, potentially inducing unnecessary surgeries. To the best of our knowledge, no previous study has systematically reviewed the extent of this overestimation.

In this study, we aimed to assess the diagnostic accuracy of highest-grade or predominant histological differentiation in T1 CRC for determining LNM. We assessed clinical utility through sensitivity and specificity. As an interpretation of these measures, increased sensitivity might reduce the rate of missed metastases, and decreased specificity could lead to unnecessary surgeries (20,21).

## METHODS

We adhered to the methodological standards of the Cochrane handbook for systematic review of diagnostic test accuracy and the preferred reporting items for systematic review and meta-analysis of diagnostic test accuracy (see PRISMA-DTA, Supplementary Digital Content 1, http://links.lww.com/CTG/B60) (19,22). Our study protocol was registered in the international prospective register of systematic reviews (PROSPERO, registration number: CRD42023416971) and on the open science framework platform (https://osf.io/TMAUN/) on April 13, 2023.

### Study selection

We included studies that assessed the impact of predominant histological differentiation and/or highest-grade differentiation in T1 CRC. We included prospective and retrospective cohort studies and case-control studies and excluded case reports, case series, reviews, meta-analyses, and duplicate cases. Patients older than 18 years with CRC who had undergone endoscopic or surgical resection were included. The exclusion criteria were familial adenomatous polyposis, Lynch syndrome, inflammatory bowel disease, preoperative chemotherapy, and radiotherapy. The index evaluated in this review was predominant histological differentiation or highest-grade differentiation. The target condition was LNM in patients with T1 CRC. The reference standard was the presence of LNM in surgical specimens. The primary outcome was diagnostic accuracy, including sensitivity and specificity, of LNM prediction in T1 CRC, as determined by predominant histological differentiation or highest-grade differentiation.

### Search method

The Cochrane Central Register of Controlled Trials (CENTRAL), MEDLINE (PubMed), and EMBASE (ProQuest Dialog) databases were searched (see Search Strategy, Supplementary Digital Content 2, http://links.lww.com/CTG/B61). In addition, we searched the World Health Organization International Clinical Trials Platform Search Portal and ClinicalTrials.gov (see Search Strategy, Supplementary Digital Content 2, http://links.lww.com/CTG/B61) for ongoing trials. We did not apply restrictions on the observation period, publication year, language, or country. We considered all studies, including published articles, conference abstracts, and letters. We checked the reference lists of all included studies, international guidelines (3–7), and articles citing eligible studies. We contacted the authors of original studies for additional data.

### Data management and assessment of methodological quality

Two of the 3 independent reviewers (J.W. and S.M. or A.M.) screened the titles and abstracts and subsequently assessed the eligibility of full-text articles. Two of the 3 independent reviewers (J.W. and S.M. or A.M.) performed independent data extraction from the included studies. Disagreements between 2 reviewers were resolved by discussion, and a third reviewer acted as an arbiter if the discussion failed (K.I. or Y.K.). We contacted the original authors if relevant data were missing. Two independent reviewers (J.W. and A.M.) evaluated the risk of bias independently using the quality assessment of diagnostic accuracy studies–2 tool (23). Disagreements between reviewers were discussed, and if agreement could not be reached, a third reviewer (K.I. or Y.K.) arbitrated.

### Data synthesis and statistical analyses

We performed a single-group analysis to examine the proportion of positive LNM in T1 CRC. We used the Metaprop command in Stata to calculate the pooled proportions with 95% confidence intervals (CIs), with inverse variance weights obtained from random-effects models (24).

Data for 2 × 2 tables of LNM against the reference standard were extracted from each study. For each of the highest-grade and predominant histological differentiations, we estimated sensitivity and specificity with 95% CIs for LNM per study using forest plots to inspect between‐study variability. In the meta-analysis, we simplified the model to a univariate random-effects logistic regression model to pool sensitivity and specificity with 95% CI separately because we were unable to fit a bivariate model owing to sparse data, few studies, or minimal observed variability in specificity (19,25). The model accounted for both within-study and between-study variability in test performance, including random effects (26). To elucidate the influence of effect modifiers on results, we performed subgroup analysis according to region (Japan vs other countries) for the highest-grade and predominant histological differentiations.

Based on the Cochrane handbook, we did not perform univariate tests for sensitivity and specificity or calculate estimates of the *I*^2^ statistic; these methods do not account for heterogeneity attributable to phenomena such as positive threshold effects (19). We performed the following sensitivity analyses to assess whether results were robust enough for the conclusions drawn in the review; only studies with participants who were surgically treated (surgical resection only and/or additional surgical resection after endoscopic resection) and those with participants who met our inclusion/exclusion criteria were included. We did not examine publication bias for the diagnostic accuracy of LNM owing to a lack of appropriate statistical methods (19).

Assuming that there were no biases, including confounding, selection, or misclassification of exposure, we simulated a patient population with T1 CRC considering 2 potential outcomes: positive LNM necessitating surgery and negative LNM not necessitating surgery. With a positive lymph node proportion of 11.2%, according to a recent systematic review (27), the simulation used 1,000 bootstrap resampling, thereby assessing the sensitivity and specificity of the highest-grade and predominant histological differentiations (28,29). This simulation enabled determining the proportion and range of false negatives (missed positive LNM) and false positives (unnecessary surgeries) when comparing the 2 differentiation classifications. We set the sensitivity and specificity of the highest grade of differentiation to the mean values obtained in the meta-analysis. In addition, we calculated the mean and range of the differences in false positives and negatives between the highest-grade and predominant differentiations in Japan and other countries.

We used STATA SE16 (version 16.1; Stata, College Station, TX) with the Metaprop, metandi, and midas packages (24,30,31) and R version 4.1.2 (The R Foundation for Statistical Computing, Vienna, Austria) with the R packages meta version 6.2.1 and boot version 1.3.28 (32,33).

## RESULTS

### Study selection and characteristics

**Figure 1.**
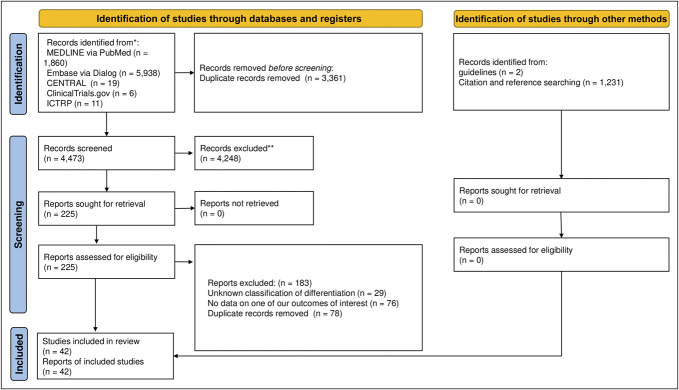
Flowchart of the study selection process.

Figure 1 illustrates the study selection process. We identified 4,473 records from 5 electronic databases after removing duplicate records on April 14, 2023. After screening, we ultimately identified 42 studies with 41,290 patients (10–14,16,34–69).

Supplementary Table 1 (see Supplementary Digital Content 4, http://links.lww.com/CTG/B63) outlines the characteristics of the studies included in this review. Among the 42 studies, 27 evaluated the diagnostic accuracy of the highest-grade differentiation for LNM in patients with T1 CRC, 14 assessed predominant histological differentiation, and 1 reported outcomes for both classifications. For highest-grade differentiation, 18 studies were from Asia and 10 from Europe and the United States. Regarding predominant differentiation, 12 studies were from Japan and 3 were from Europe and Asia, excluding Japan. In addition, one study from Japan addressed both classifications.

**Figure 2.**
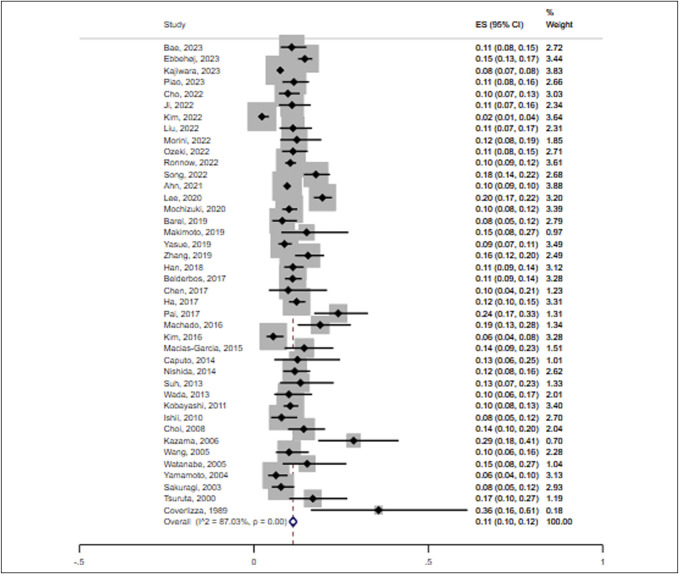
Forest plot for the proportion of lymph node metastasis in T1 colorectal cancer. ES, effect size; CI, confidence interval.

Supplementary Digital Content 3 (http://links.lww.com/CTG/B62) displays the risk of bias assessment of the eligible studies using the quality assessment of diagnostic accuracy studies–2 tool. For the domain of patient selection, we scored 1 study as having an unclear risk of bias and 8 as having high applicability concerns. Regarding the index test, all 42 studies had an unclear risk of bias with low applicability concerns. Regarding the reference standard, 3 studies had a high risk of bias and 8 had high applicability concerns. In the flow and timing domain, 8 studies had a high risk of bias. The pooled proportion of LNM in T1 CRC was 11% (95% CI 10%–12%) (Figure 2).

### Diagnostic accuracy of highest-grade or predominant histological differentiation for LNM

**Figure 3.**
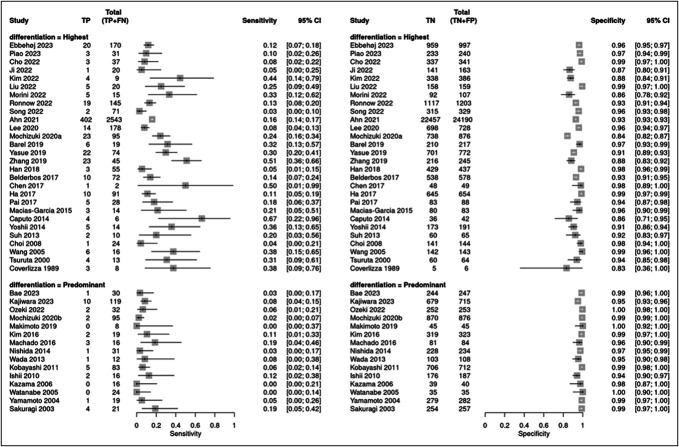
Forest plot showing the sensitivity and specificity of the highest-grade and predominant histological differentiations for prediction of lymph node metastasis. CI, confidence interval.

Figure 3 shows the analysis of the differentiation classifications. They had pooled sensitivities of 0.18 (95% CI 0.13–0.24) and 0.06 (95% CI 0.04–0.09) (test for subgroup differences, *P* < 0.0001) and specificities of 0.95 (95% CI 0.93–0.96) and 0.98 (95% CI 0.97–0.99) (test for subgroup differences, *P* < 0.0001) for the highest-grade and predominant histological differentiations, respectively.

In Japan, differentiation classification had pooled sensitivities of 0.28 (95% CI 0.22–0.34) and 0.06 (95% CI 0.04–0.09) (test for subgroup differences, *P* < 0.0001) and specificities of 0.89 (95% CI 0.85–0.92) and 0.98 (95% CI 0.97–0.99) (test for subgroup differences, *P* < 0.0001) for the highest-grade and predominant histological differentiations, respectively.

In other countries, differentiation classification had pooled sensitivities of 0.16 (95% CI 0.12–0.23) and 0.09 (95% CI 0.04–0.19) (test for subgroup differences, *P* = 0.167) and specificities of 0.96 (95% CI 0.94–0.97) and 0.98 (95% CI 0.97–0.99) (test for subgroup differences, *P* = 0.0029) in the highest-grade and predominant histological differentiations, respectively.

### Additional analyses

**Figure 4.**
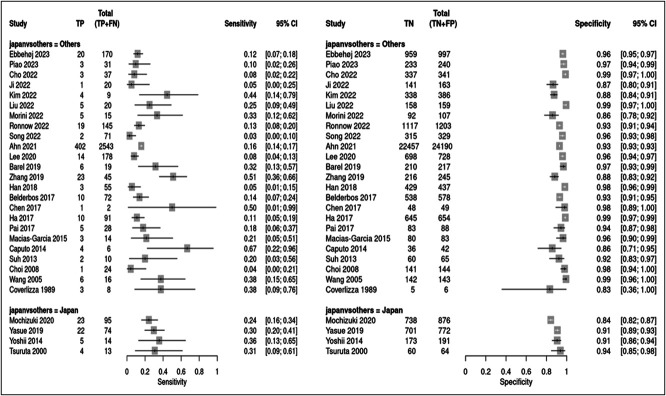
Forest plot for the subgroup analysis according to region (Japan and other countries) regarding highest-grade differentiation. CI, confidence interval.

Figure 4 shows the results of subgroup analysis by region (Japan and other countries); highest-grade differentiation had pooled sensitivities of 0.28 (95% CI 0.22–0.34) and 0.16 (95% CI 0.12–0.23) (test for subgroup differences, *P* = 0.011) and specificities of 0.89 (95% CI 0.85–0.92) and 0.96 (95% CI 0.94–0.97) (test for subgroup differences, *P* = 0.0003) in Japan and other countries, respectively.

**Figure 5.**
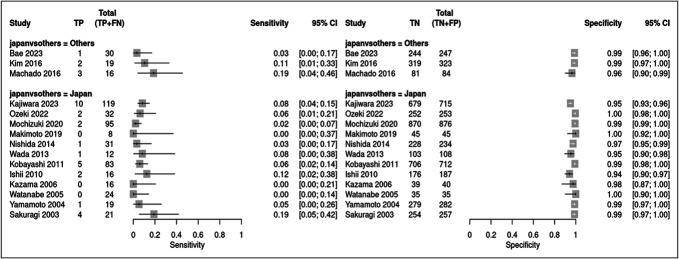
Forest plot for the subgroup analysis according to region (Japan and other countries) regarding predominant differentiation. CI, confidence interval.

Figure 5 shows the results of subgroup analysis by region (Japan and other countries); predominant differentiation had pooled sensitivities of 0.06 (95% CI 0.04–0.08) and 0.09 (95% CI 0.04–0.21) (test for subgroup differences, *P* = 0.28) and specificities of 0.98 (95% CI 0.97–0.99) and 0.98 (95% CI 0.97–0.99) (test for subgroup differences, *P* = 0.93) in Japan and other countries, respectively.

The results of sensitivity analyses were consistent with the main results (see Supplementary Table 2, Supplementary Digital Content 5, http://links.lww.com/CTG/B64). In the bootstrap simulation, the difference in sensitivity between the highest-grade and predominant differentiations was 0.12 (95% CI 0.060–0.18), and the difference in specificity was −0.034 (95% CI −0.018 to −0.054). In the simulated cohort of 100 patients with T1 CRC, with an assumed LNM proportion of 11.2%, the differences in false positives and negatives between the highest-grade and predominant differentiations were 3.0 individuals (range 1.6–4.4) and −1.3 individuals (range −2.0 to −0.7), respectively.

In Japan, in the bootstrap estimation, the difference in sensitivity between the highest grade and predominant differentiations was 0.21 (95% CI 0.17–0.27) and that in specificity was −0.091 (95% CI −0.14 to −0.064). In the simulated cohort of 100 patients with T1 CRC, with an assumed LNM proportion of 11.2%, the difference in false positives and negatives between the highest-grade and predominant differentiations was 8.1 individuals (range 5.7–9.8) and −2.3 individuals (range −3.0 to −1.9), respectively.

In other countries, in the bootstrap estimation, the difference in sensitivity between the highest-grade and predominant differentiations was 0.066 (95% CI −0.025 to 0.16), and the difference in specificity was −0.029 (95% CI −0.047 to −0.014). In the simulated cohort of 100 patients with T1 CRC, with an assumed LNM proportion of 11.2%, the differences in false positives and negatives between the highest-grade and predominant differentiations were 2.6 individuals (range 1.3–3.6) and −0.7 individuals (range −2.0 to 0.05), respectively.

## DISCUSSION

The systematic review and meta-analysis of 42 studies involving 41,290 patients investigated the diagnostic accuracy of highest-grade and predominant histological differentiations in T1 CRC for predicting LNM. We found that the sensitivity of highest-grade differentiation was higher than that of predominant histological differentiation, whereas the specificity of the latter was higher than that of the former. In the 100 patients with T1 CRC, using highest-grade differentiation could have prevented oversight of 1 case with LNM, whereas using predominant differentiation could have prevented additional surgeries in 3 patients.

PD was found to have high sensitivity, especially with highest-grade differentiation, and low specificity, especially with predominant differentiation. International guidelines recommend evaluating 4 predictive factors for LNM (PD, depth of submucosal invasion, LVI, and BD) before deciding on additional surgical resection following endoscopic resection. In previous studies, depth of submucosal invasion, LVI, and BD showed sensitivities of 0.95, 0.25, and 0.25 and specificities of 0.40, 0.94, and 0.95, respectively (40,60,70). Compared with other major predictive factors, PD might have the highest specificity but the lowest sensitivity. Given that the specificity of PD is sufficiently high, adopting the highly sensitive high-grade differentiation classification over predominant differentiation as a stand-alone predictor may help prevent misclassification owing to increased sensitivity. Contrarily, previous studies have shown the sensitivity of all 4 predictive factors combined to positive LNM to be 100%, whereas specificity was extremely low (21,71). When considering all 4 factors, adopting the highly specific predominant differentiation classification might lead to increased specificity of the overall predictors.

The choice of highest-grade or predominant differentiation classification hinges on multiple factors, such as patient characteristics and the health care economic circumstances of the country or facility. To illustrate this, selection of the highest-grade differentiation might be more beneficial for patients with certain attributes, including those at low surgical risk (younger patients or those without comorbidities) who wish to survive longer (72,73) because highest-grade differentiation was more effective than predominant differentiation in preventing 1% of missed lymph node–positive patients from undergoing surgery. LNM-positive patients can have potentially fatal outcomes, including metastatic recurrence, if not treated surgically. In addition, the economic situation of the country can significantly influence this choice. In a country with high health care costs or high surgical risk, choosing predominant differentiation could be more cost-effective, providing similar benefits at a lower cost, because additional surgeries could impose a high cost of approximately $8,000 USD, besides being associated with the risk of surgery-related mortality (1.7%) and recurrence (0.7%–1.0%) despite tumor resection (72,74–76). Furthermore, older patients with multiple comorbidities and patients with lower rectal cancer, despite the high risk of LNM, may be candidates for observation rather than for surgery (77). The benefits of surgical resection, especially in older patients and those with severe comorbidities, may be limited owing to its impact on prognosis (78). In addition, for patients with lower rectal tumors, surgery (specifically lymph node dissection) carries the risk of inducing anal, urinary, and sexual dysfunction (79,80). When comparing highest-grade differentiation with predominant differentiation, the choice cannot be made in isolation. Individual patient needs and economic realities need to be weighed in this multifaceted and complex decision, which can have far-reaching implications for patient health outcomes and economic sustainability of the health care system. Hence, an informed decision that balances both considerations is crucial. Thus, selection of highest-grade differentiation or predominant differentiation is not one-size-fits-all, but must be discussed by clinicians, patients, pathologists, and policymakers using the Delphi method according to international guidelines in each country and considering individual and societal factors (81).

We recommend that pathologists assessing cases of T1 CRC use both classification systems and provide information about highest-grade and predominant differentiation. In addition, in cases where a cancerous lesion is associated with multiple coexisting histologic types, it is beneficial to list each histological type in order of area predominance, as outlined in the Japanese guidelines (3). Furthermore, the overall proportion of PD can aid in identifying PD possibly missed in subsequent histological sections. A comprehensive approach incorporating both classifications can provide valuable information for well-informed clinical decisions. This inclusive evaluation ensures better understanding of the differentiation status of the tumor, facilitating the tailoring of treatment strategies and the most appropriate management approach for each patient.

Variations in guideline recommendations for differentiation classification across countries may have contributed to differences in the assessment protocols for histopathological diagnosis and the observed variability in diagnostic accuracy between Japan and other countries in subgroup analysis (3,4,6,8). These disparities could be attributed to differences in the handling and processing of excised specimens, variations in histological evaluation among pathologists with varying levels of expertise and experience, and diverse differentiation classification criteria across countries (3,4,6,8). To ensure consistent and standardized interpretation of diagnostic criteria, it is crucial to establish uniform international diagnostic criteria for differentiation classification in cases of CRC. Such guidelines would facilitate harmonization of practice among pathologists worldwide and improve the reliability and comparability of diagnostic accuracy assessments across different regions.

This study has some limitations. First, considerable variation exists in the analysis and reporting of histological factors, including differentiation classification, the diagnostic concordance rate of histology, and the implementation rate of immunostaining (82) across facilities and countries. In particular, the diagnostic concordance rate between Japanese pathologists and those in Asia, Europe, and the United States is a major problem. However, we investigated specific diagnostic criteria for differentiation and evaluated cross-country differences using subgroup analysis. There is a need to unify tumor grading criteria as a future global initiative. Second, the specific threshold for the proportion of PD that predicts LNM in cases of differentiated cancer with mixed PD components remains unknown. Therefore, further studies should focus on determining this threshold in T1 CRC. Third, the lack of multivariable analysis in our meta-analysis of included studies prevented the determination of potential interactions between different predictive factors for LNM. Caution should be exercised when interpreting results from a meta-analysis of multivariable studies. This is because of inconsistencies in the classification of differentiation for other predictive factors, such as the evaluation of the depth of submucosal invasion or LVI with or without additional staining. Although an earlier meta-analysis of multivariable studies had reported depth of submucosal invasion as an inadequate predictor of LNM (27), one study in the meta-analysis used predominant differentiation and reported depth of submucosal invasion to be associated with positive LNM. A recent multicenter study using predominant differentiation reported depth of submucosal invasion as an independent predictor for LNM (49). The outcomes may differ for these factors because of the varying evaluation methods across the studies. Further studies are needed to evaluate the classification of differentiation, adjusted for other predictive factors, through meta-analysis or multinational and multicenter studies of individual patient data. Last, it is problematic to evaluate the same data for endoscopically treated cases in Japan, where endoscopic submucosal dissection is usually performed as a batch resection, and in Europe and the United States, where segmental resection is more common. However, we evaluated cross-country differences using subgroup analysis.

In conclusion, this systematic review and meta-analysis demonstrated that highest-grade differentiation had greater sensitivity than predominant histological differentiation, while predominant histological differentiation exhibited greater specificity than highest-grade differentiation. Applying highest-grade differentiation could avert missing 1% of LNM, while using predominant differentiation could prevent 3% of unnecessary surgeries. The findings overall implied that clinicians, patients, pathologists, and policymakers should discuss the choice of highest-grade and predominant differentiations, balancing patient health outcomes and economic sustainability of the health care system. Further studies are needed to evaluate differentiation classification adjusted for other predictive factors through individual patient data meta-analyses or multinational and multicenter studies.

## CONFLICTS OF INTEREST

**Guarantor of the article:** Jun Watanabe, MD, PhD.

**Specific author contributions:** J.W., K.I., and Y.K.: contributed to the study concept and design and drafting of the manuscript. J.W.: obtained funding. J.W., K.I., Y.K., S.M., A.M., and K.G.Y.: contributed to the statistical analysis and interpretation of data. K.K. and N.S.: contributed to administrative support and study supervision. J.W., K.I., and Y.K.: developed the software. J.W., K.I., Y.K., S.M., A.M., K.G.Y., F.M.d.J., and I.M.: contributed to data collection and critical revision of the manuscript. S.K., K.K., and N.S.: contributed to critical revision of the manuscript.

**Financial support:** The study was supported by JSPS KAKENHI (grant number JP21K21121 and 23K16289).

**Potential competing interests:** None to report.

## Supplementary Material
