# Supplementary material for: Diagnostic Accuracy of Highest-Grade or Predominant Histological Differentiation of T1 Colorectal Cancer in Predicting Lymph Node Metastasis: A Systematic Review and Meta-Analysis
Source: Clin Transl Gastroenterol. 2024 Jan 2;15(3):e00673. doi: 10.14309/ctg.0000000000000673 (PMC10962900; doi:10.14309/ctg.0000000000000673)
Supplement: Supplementary file 3 [file ct9-15-e00673-s003.docx]

**Search Strategy, Supplemental Digital Content 2**

**CENTRAL search strategy**

#1. [mh "Colorectal Neoplasms"]

#2. ((colorect*:ti,ab OR colon*:ti,ab OR rect*:ti,ab) NEAR/1 (carcinoma*:ti,ab OR neoplas*:ti,ab OR adenocarcinom*:ti,ab OR cancer*:ti,ab OR tumor*:ti,ab OR tumour*:ti,ab OR malignan*:ti,ab))

#3. #1 OR #2

#4. (T1:ti,ab OR pT1:ti,ab OR submucosal:ti,ab OR early:ti,ab)

#5. #3 AND #4

#6. [mh “Lymphatic Metastasis”]

#7. (lymph*:ti,ab adj2 metasta*:ti,ab)

#8. #6 OR #7

#9. #5 AND #8

**MEDLINE (via Ovid) search strategy**

1 exp Colorectal Neoplasms/

2 ((colorect* or colon* or rect*) adj1 (carcinoma* or neoplas* or adenocarcinom* or cancer* or tumor* or tumour* or malignan*)).tw.

3 1 or 2

4 (T1 or pT1 or submucosal or early).tw.

5 3 and 4

6 exp Lymphatic Metastasis/

7 (lymph* adj2 metasta*).tw.

8 6 or 7

9 5 and 8

**EMBASE (via ProQuest Dialog) search strategy**

S1 (EMB.EXACT.EXPLODE("colorectal tumor"))

S2 ((ti(colorect*) or ti(colon*) or ti(rect*)) AND (ti(carcinoma*) or ti(neoplas*) or ti(adenocarcinom*) or ti(cancer*) or ti(tumor*) or ti(tumour*) or ti(malignan*))) OR ((ab(colorect*) or ab(colon*) or ab(rect*)) AND (ab(carcinoma*) or ab(neoplas*) or ab(adenocarcinom*) or ab(cancer*) or ab(tumor*) or ab(tumour*) or ab(malignan*)))

S3 S1 OR S2

S4 (ti(T1) or ti(pT1) or ti(submucosal) or ti(early)) OR (ab(T1) or ab(pT1) or ab(submucosal) or ab(early))

S5 S3 AND S4

S6 (EMB.EXACT.EXPLODE("lymphatic system metastasis"))

S7 (ti(lymph*) and ti(metasta*)) OR (ab(lymph*) and ab(metasta*))

S8 S6 OR S7

S9 S5 AND S8

**ICTRP search strategy**

(((colorectal or colon or rectum) AND (carcinoma or carcinomas or neoplasm or neoplasms or adenocarcinoma or adenocarcinomas or cancer or cancers or tumor or tumors or tumour or tumours or malignancy or malignancies)) AND (T1 or pT1 or submucosal or early)) AND (lymph metastasis)

**ClinicalTrials.gov search strategy**

Condition or disease: ((colorectal OR colon OR rectum) AND (carcinoma OR carcinomas OR neoplasm OR neoplasms OR adenocarcinoma OR adenocarcinomas OR cancer OR cancers OR tumor OR tumors)) AND (T1 OR pT1 OR submucosal OR early)

Other terms: lymph metastasis
