# Supplementary material for: Diagnostic Accuracy of Highest-Grade or Predominant Histological Differentiation of T1 Colorectal Cancer in Predicting Lymph Node Metastasis: A Systematic Review and Meta-Analysis
Source: Clin Transl Gastroenterol. 2024 Jan 2;15(3):e00673. doi: 10.14309/ctg.0000000000000673 (PMC10962900; doi:10.14309/ctg.0000000000000673)
Supplement: Supplementary file 4 [file ct9-15-e00673-s004.docx]

**The modified Quality Assessment of Diagnostic Accuracy Studies-2 (QUADAS-2) tool, Supplemental Digital Content 3**

**Risk of Bias**

Patient Selection

Was a consecutive or random sample of patients enrolled?

Was a case–control design avoided?

Did the study avoid inappropriate exclusions?

We will judge participants based on our inclusion/exclusion criteria.

Index Text

Were the index test results interpreted without knowledge of the results of the reference standard?

Reference Standard

Is the reference standard likely to correctly classify the target condition?

We will judge D0 dissection surgery inappropriate.

Were the reference standard results interpreted without knowledge of the results of the index test?

Flow and Timing

Was there an appropriate interval between the index test and reference standard?

Did all patients receive the same reference standard?

Were all patients included in the analysis?

**Applicability**

Patient Selection

Are There Concerns That the Included Patients and Setting Do Not Match the Review Question?

Index Test

Are There Concerns That the Index Test, Its Conduct, or Its Interpretation Differ from the Review Question?

Reference Standard

Are There Concerns That the Target Condition as Defined by the Reference Standard Does Not Match the Question?

| Authors, year | Risk of bias | | | | Applicability concerns | | |
| --- | --- | --- | --- | --- | --- | --- | --- |
|  | Patient selection | Index test | Reference standard | Flow and timing | Patient selection | Index test | Reference standard |
| Bae, 2023 | low | unclear | low | low | low | low | low |
| Ebbehøj, 2023 | low | unclear | low | low | low | low | low |
| Kajiwara, 2023 | low | unclear | low | high | high | low | high |
| Piao, 2023 | low | unclear | low | high | high | low | high |
| Cho, 2022 | low | unclear | low | high | high | low | high |
| Ji, 2022 | low | unclear | low | low | low | low | low |
| Kim, 2022 | low | unclear | low | low | low | low | low |
| Liu, 2022 | low | unclear | low | low | low | low | low |
| Morini, 2022 | low | unclear | low | low | low | low | low |
| Ozeki, 2022 | low | unclear | low | low | low | low | low |
| Ronnow, 2022 | low | unclear | low | low | low | low | low |
| Song, 2022 | low | low | low | low | low | low | low |
| Ahn, 2021 | unclear | unclear | high | high | high | low | high |
| Lee, 2020 | low | unclear | low | low | low | low | low |
| Mochizuki, 2020 | low | unclear | low | low | low | low | low |
| Barel, 2019 | low | unclear | high | high | high | low | high |
| Makimoto, 2019 | low | low | low | low | low | low | low |
| Yasue, 2019 | low | unclear | low | low | low | low | low |
| Zhang, 2019 | low | unclear | low | low | low | low | low |
| Han, 2018 | low | unclear | low | low | low | low | low |
| Belderbos, 2017 | low | unclear | low | low | low | low | low |
| Chen, 2017 | low | low | low | low | low | low | low |
| Ha, 2017 | low | unclear | low | low | low | low | low |
| Pai, 2017 | low | unclear | low | low | low | low | low |
| Machado, 2016 | low | unclear | high | high | high | low | high |
| Kim, 2016 | low | unclear | low | low | low | low | low |
| Macias-Garcia, 2015 | low | unclear | low | low | low | low | low |
| Caputo, 2014 | low | unclear | low | low | low | low | low |
| Nishida, 2014 | low | unclear | low | low | low | low | low |
| Yoshii, 2014 | low | unclear | high | high | high | low | high |
| Suh, 2013 | low | low | low | low | low | low | low |
| Wada, 2013 | low | unclear | low | low | low | low | low |
| Kobayashi, 2011 | low | unclear | low | low | low | low | low |
| Ishii, 2010 | low | unclear | low | low | low | low | low |
| Choi, 2008 | low | unclear | low | low | low | low | low |
| Kazama, 2006 | low | unclear | low | low | low | low | low |
| Wang, 2005 | low | unclear | low | low | low | low | low |
| Watanabe, 2005 | low | unclear | low | low | low | low | low |
| Yamamoto, 2004 | low | unclear | low | high | high | low | high |
| Sakuragi, 2003 | low | unclear | low | high | high | low | high |
| Tsuruta, 2000 | low | unclear | low | low | low | low | low |
| Coverlizza, 1989 | low | low | low | low | low | low | low |

Risk of bias and applicability were rated on seven domains using the modified Quality Assessment of Diagnostic Accuracy Studies-2 tool by two of the four reviewers (JW and MA, HS, or MH). The four domains assessed for bias were “Patient selection,” “Index test,” “Reference standard,” and “Flow and timing,” and the three domains assessed for applicability were “Patient selection,” “Index test,” and “Reference standard.” Each risk of bias and applicability domain was classified as high, low, or unclear according to prespecified signaling questions.
