# Supplementary material for: Diagnostic Accuracy of Highest-Grade or Predominant Histological Differentiation of T1 Colorectal Cancer in Predicting Lymph Node Metastasis: A Systematic Review and Meta-Analysis
Source: Clin Transl Gastroenterol. 2024 Jan 2;15(3):e00673. doi: 10.14309/ctg.0000000000000673 (PMC10962900; doi:10.14309/ctg.0000000000000673)
Supplement: Supplementary file 5 [file ct9-15-e00673-s005.docx]

**Supplemental Table 1.** Overview of baseline characteristics

| Authors, year | Country | Study design | Recruitment period | Definition of differentiation (highest grade or predominant) | Total patients, n | Males, n | Age (years) | Total number of LNM, n (%) | Rate of unfavorable differentiation, n (%) | Definition of differentiation | Treatment method |
| --- | --- | --- | --- | --- | --- | --- | --- | --- | --- | --- | --- |
| Bae, 2023 | Korea | Retrospective study | 2000-2015 | Predominant grade by JSCCR classification 2019 | 277 | 148 | 60 | 30 (10.8) | 4 (1.4) | Por | Primary and additional surgery |
| Ebbehøj, 2023 | Denmark | Retrospective study | 2016-2019 | Highest grade by WHO | 1167 | 639 | 68 | 170 (14.6) | 58 (5.0) | Por | Primary and additional surgery |
| Kajiwara, 2023 | Japan | Retrospective study | 2009-2016 | Predominant grade by JSCCR classification | 4673 | 2770 | 67 | 352 (8.1) | 59 (1.3) | Por, sig, muc | Primary and additional surgery, and endoscopy |
| Piao, 2023 | China | Retrospective study | 2015-2021 | Highest grade by WHO | 271 | 168 | 65 | 31 (11.4) | 10 (3.7) | Por | Primary and additional surgery, and endoscopy |
| Cho, 2022 | Korea | Retrospective study | 2010-2020 | Highest grade by WHO | 380 | 232 | 66 | 37 (9.7) | 7 (1.8) | Por, muc | Primary and additional surgery, and endoscopy |
| Ji, 2022 | China | Retrospective study | 2002-2020 | Highest grade by WHO | 183 | 112 | NA | 20 (10.9) | 23 (12.6) | Por | Primary surgery |
| Kim, 2022 | Korea | Retrospective study | 2002-2019 | Highest grade by WHO | 395 | 225 | 63 | 9 (2.3) | 52 (13.2) | Por, sig, muc, undifferentiated | Primary and additional surgery |
| Liu, 2022 | China | Retrospective study | 2010-2020 | Highest grade by WHO | 179 | 103 | 60 | 20 (11.1) | 6 (3.4) | Por, sig, muc, NEC | Primary and additional surgery |
| Morini, 2022 | Italy | Retrospective study | 2000-2018 | Highest grade by AJCC Cancer Staging Manual 8th edition | 122 | 65 | NA | 15 (12.3) | 20 (16.4) | Por | Primary and additional surgery |
| Ozeki, 2022 | Japan | Retrospective study | 2003-2019 | Predominant grade by JSCCR classification 2019 | 285 | 154 | 69 | 32 (11.2) | 3 (1.1) | Por, sig, muc | Primary and additional surgery |
| Ronnow, 2022 | Sweden/ Denmark | Prospective study | 2009-2017/ 2016-2018 | Highest grade by WHO | 1439 | 752 | 71 | 150 (10.4) | 145 (9.7) | Por | Primary and additional surgery |
| Song, 2022 | Korea | Retrospective study | 2010-2018 | Highest grade by WHO | 400 | 239 | 59 | 71 (17.8) | 16 (4.0) | Por | Additional surgery |
| Ahn, 2021 | USA | Retrospective study | 2004-2016 | Highest grade by AJCC Cancer Staging Manual 7th edition | 26733 | 14118 | NA | 2543 (9.5) | 2135 (8.0) | Por, undifferentiated | NA |
| Lee, 2020 | Korea | Retrospective study | 2008-2013 | Highest grade by AJCC Cancer Staging Manual | 906 | 532 | 60 | 178 (19.6) | 44 (4.9) | Por, sig, muc, undifferentiated | Primary and additional surgery |
| Mochizuki, 2020 | Japan | Retrospective study | 2001-2018 | Highest grade by WHO | 745 | NA | NA | 75 (10.1) | 149 (13.0) | Por, muc | Primary and additional surgery |
| Barel, 2019 | France | Retrospective study | 2009-2013 | Highest grade by WHO | 234 | 134 | 67 | 19 (8.1) | 13 (5.6) | Por, undifferentiated | Primary and additional surgery, and endoscopy |
| Makimoto, 2019 | Japan | Retrospective study | 2010-2018 | Predominant grade by JSCCR classification 2010 and 2016 | 53 | 24 | 68 | 8 (15.1) | 0 (0) | Por, sig, muc | Additional surgery |
| Yasue, 2019 | Japan | Retrospective study | 2005-2016 | Highest grade by WHO | 846 | 470 | 66 | 74 (8.7) | 93 (11.0) | Por, sig, muc | Primary and additional surgery, and endoscopy |
| Zhang, 2019 | China | Retrospective study | 2008-2014 | Highest grade by WHO | 290 | 151 | 60 | 45 (15.5) | 52 (17.9) | Por | Primary and additional surgery |
| Han, 2018 | Korea | Retrospective study | 2008-2012 | Highest grade by WHO | 492 | 296 | 61 | 55 (11.2) | 11 (2.2) | Por, muc | Primary and additional surgery |
| Belderbos, 2017 | Netherlands | Retrospective study | 1995-2011 | Highest grade by AJCC Cancer Staging Manual 7th edition | 650 | NA | NA | 72 (11.1) | 50 (7.7) | Por, undifferentiated | Primary and additional surgery |
| Chen, 2017 | China | Retrospective study | 2007-2013 | Highest grade by WHO | 51 | 31 | 63 | 5 (9.8) | 2 (3.9) | Por | Additional surgery |
| Ha, 2017 | Korea | Retrospective study | 2001-2015 | Highest grade by WHO | 745 | 471 | 61 | 91 (12.2) | 19 (2.6) | Por | Primary and additional surgery |
| Pai, 2017 | USA | Retrospective study | 2010-2014 | Highest grade by WHO | 116 | 62 | 63 | 28 (24.1) | 10 (8.6) | Por | Primary and additional surgery |
| Machado, 2016 | Spain | Retrospective study | 2006-2014 | Predominant grade by the College of American Pathologist protocol classification | 100 | NA | NA | 19 (19.0) | 6 (6.0) | Por, sig, muc | Primary and additional surgery |
| Kim, 2016 | Korea | Retrospective study | 2005-2012 | Predominant grade by JSCCR classification 2010 | 344 | NA | NA | 19 (5.5) | 6 (1.7) | Por | Primary and additional surgery |
| Macias-Garcia, 2015 | Spain | Retrospective study | 2000-2011 | Highest grade by WHO | 97 | 61 | 68 | 14 (14.4) | 6 (6.2) | Por | Primary and additional surgery |
| Caputo, 2014 | Italy | Retrospective study | 2001-2013 | Highest grade by WHO | 48 | 23 | 71 | 6 (12.5) | 10 (20.8) | Por | Primary and additional surgery |
| Nishida, 2014 | Japan | Retrospective study | 2000-2011 | Predominant grade | 265 | 161 | 65 | 31 (11.7) | 7 (2.6) | Por | Primary surgery |
| Yoshii, 2014 | Japan | Retrospective study | 1989-2008 | Highest grade by WHO | 205 | NA | NA | 14 (6.8) | 23 (11.2) | Por, sig, muc | Additional surgery and endoscopy |
| Suh, 2013 | Korea | Prospective study | 2007-2012 | Highest grade by WHO | 75 | NA | NA | 10 (13.3) | 7 (9.3) | Por | Additional surgery |
| Wada, 2013 | Japan | Retrospective study | 1995-2005 | Predominant grade | 120 | 82 | 64 | 12 (10.0) | 6 (5.0) | Por | Primary and additional surgery |
| Kobayashi, 2011 | Japan | Retrospective study | 1991-1996 | Predominant grade by JSCCR classification | 798 | 379 | 60 | 83 (10.4) | 11 (1.4) | Por, muc | Primary and additional surgery |
| Ishii, 2010 | Japan | Retrospective study | 25 years | Predominant grade | 203 | NA | NA | 16 (7.9) | 13 (6.4) | Por | Primary and additional surgery |
| Choi, 2008 | Korea | Retrospective study | 1989-2004 | Highest grade by WHO | 168 | 99 | 57 | 24 (14.3) | 4 (2.4) | Por | Primary and additional surgery |
| Kazama, 2006 | Japan | Retrospective study | 1990-2001 | Predominant grade by JSCCR classification | 56 | 41 | 63 | 16 (28.6) | 1 (1.8) | Por | Primary surgery |
| Wang, 2005 | Taiwan | Retrospective study | 1969-2002 | Predominant grade | 159 | 107 | 65 | 16 (10.1) | 7 (4.4) | Por | Primary and additional surgery, and endoscopy |
| Watanabe, 2005 | Japan | Retrospective study | 1997-2003 | Predominant grade | 59 | 36 | 65 | 9 (15.3) | 0 (0) | NA | Primary surgery |
| Yamamoto, 2004 | Japan | Retrospective study | 1970-2001 | Predominant grade by JSCCR classification | 301 | 218 | 62 | 19 (6.3) | 4 (1.3) | Por | Primary and additional surgery, and endoscopy |
| Sakuragi, 2003 | Japan | Retrospective study | 1979-2000 | Predominant grade by JSCCR classification | 271 | 175 | 62 | 21 (7.7) | 7 (2.6) | Por, sig, muc | Primary and additional surgery, and endoscopy |
| Tsuruta, 2000 | Japan | Retrospective study | 1995-1999 | Highest grade by WHO | 77 | 56 | 63 | 13 (16.9) | 8 (10.4) | Por, sig, muc | Primary and additional surgery |
| Coverlizza, 1989 | Italy | Retrospective study | 1975-1987 | Highest grade by WHO | 14 | 7 | 57 | 5 (35.7) | 4 (28.6) | Por | Additional surgery |

*Only cases with radical surgery or completion surgery were included. If endoscopic resection was performed without completion surgery and there was no specific statement on LNM-status, cases were excluded.

Abbreviations used in this paper: AJCC, American Joint Committee on Cancer; CI confidence interval; JCCRS, Japanese Society for Cancer of the Colon and Rectum; LNM, lymph node metastasis; NA, not applicable; NEC, neuroendocrine carcinoma; WHO, World Health Organization.

**References**

1 Bae HJ, Ju H, Lee HH, et al. Long-term outcomes after endoscopic versus surgical resection of T1 colorectal carcinoma. Surg Endosc 2023;37:1231-41. https://doi.org/10.1007/s00464-022-09649-1

2 Yoshii S, Nojima M, Nosho K, et al. Factors associated with risk for colorectal cancer recurrence after endoscopic resection of T1 tumors. Clin Gastroenterol Hepatol 2014;12:292-302.e3. https://doi.org/10.1016/j.cgh.2013.08.008

3 Ebbehøj AL, Smith HG, Jørgensen LN, et al. Prognostic factors for lymph node metastases in pT1 colorectal cancer differ according to tumor morphology: A nationwide cohort study. Ann Surg 2023;277:127-35. https://doi.org/10.1097/sla.0000000000005684

4 Kajiwara Y, Oka S, Tanaka S, et al. Nomogram as a novel predictive tool for lymph node metastasis in T1 colorectal cancer treated with endoscopic resection: a nationwide, multicenter study. Gastrointest Endosc 2023;97:1119-28.e5. https://doi.org/10.1016/j.gie.2023.01.022

5 Piao Z, Ge R, Wang C. A proposal for grading the risk of lymph node metastasis after endoscopic resection of T1 colorectal cancer. Int J Colorectal Dis 2023;38:25. https://doi.org/10.1007/s00384-023-04319-7

6 Cho SH, Park BS, Son GM, et al. Differences in factors predicting lymph node metastasis between pT1 rectal cancer and pT1 colon cancer: A retrospective study. Am Surg 2022:31348221111517. https://doi.org/10.1177/00031348221111517

7 Ji X, Kang M, Zhao X, et al. Poorly differentiated cluster grade-a vital predictor for lymph node metastasis and oncological outcomes in patients with T1 colorectal cancer: A retrospective study. BMC Gastroenterol 2022;22:409. https://doi.org/10.1186/s12876-022-02492-7

8 Kim JK, Rhee YY, Bae JM, et al. Composite scoring system and optimal tumor budding cut-off number for estimating lymph node metastasis in submucosal colorectal cancer. BMC Cancer 2022;22:861. https://doi.org/10.1186/s12885-022-09957-8

9 Liu Z, Huang C, Tian H, et al. Establishment of a dynamic nomogram for predicting the risk of lymph node metastasis in T1 stage colorectal cancer. Front Surg 2022;9:845666. https://doi.org/10.3389/fsurg.2022.845666

10 Morini A, Annicchiarico A, De Giorgi F, et al. Local excision of T1 colorectal cancer: Good differentiation, absence of lymphovascular invasion, and limited tumor radial infiltration (≤4.25 mm) may allow avoiding radical surgery. Int J Colorectal Dis 2022;37:2525-33. https://doi.org/10.1007/s00384-022-04279-4

11 Ozeki T, Shimura T, Ozeki T, et al. The risk analyses of lymph node metastasis and recurrence for submucosal invasive colorectal cancer: Novel criteria to skip completion surgery. Cancers 2022;14. <https://doi.org/10.3390/cancers14030822>

12 Rönnow CF, Arthursson V, Toth E, et al. Lymphovascular infiltration, not depth of invasion, is the critical risk factor of metastases in early colorectal cancer: Retrospective population-based cohort study on prospectively collected data, including validation. Ann Surg 2022;275:e148-54. https://doi.org/10.1097/sla.0000000000003854

13 Song JH, Hong Y, Kim ER, et al. Utility of artificial intelligence with deep learning of hematoxylin and eosin-stained whole slide images to predict lymph node metastasis in T1 colorectal cancer using endoscopically resected specimens; prediction of lymph node metastasis in T1 colorectal cancer. J Gastroenterol 2022;57:654-66. https://doi.org/10.1007/s00535-022-01894-4

14 Ahn JH, Kwak MS, Lee HH, et al. Development of a novel prognostic model for predicting lymph node metastasis in early colorectal cancer: Analysis based on the surveillance, epidemiology, and end results database. Front Oncol 2021;11:614398. https://doi.org/10.3389/fonc.2021.614398

15 Lee YJ, Huh JW, Shin JK, et al. Risk factors for lymph node metastasis in early colon cancer. Int J Colorectal Dis 2020;35:1607-13. https://doi.org/10.1007/s00384-020-03618-7

16 Mochizuki K, Kudo S-E, Ichimasa K, et al. Left-sided location is a risk factor for lymph node metastasis of T1 colorectal cancer: a single-center retrospective study. Int J Colorectal Dis 2020;35:1911-9. https://doi.org/10.1007/s00384-020-03668-x

17 Barel F, Cariou M, Saliou P, et al. Histopathological factors help to predict lymph node metastases more efficiently than extra-nodal recurrences in submucosa invading pT1 colorectal cancer. Sci Rep 2019;9:8342. https://doi.org/10.1038/s41598-019-44894-w

18 Makimoto S, Takami T, Hatano K, et al. Additional surgery after endoscopic submucosal dissection for colorectal cancer: a review of 53 cases. Int J Colorectal Dis 2019;34:1723-9. https://doi.org/10.1007/s00384-019-03370-7

19 Yasue C, Chino A, Takamatsu M, et al. Pathological risk factors and predictive endoscopic factors for lymph node metastasis of T1 colorectal cancer: a single-center study of 846 lesions. J Gastroenterol 2019;54:708-17. https://doi.org/10.1007/s00535-019-01564-y

20 Zhang Q, Wang L, Huang D, et al. Pathological risk factors for lymph node metastasis in patients with submucosal invasive colorectal carcinoma. Cancer Manag Res 2019;11:1107-14. https://doi.org/10.2147/cmar.s181740

21 Han J, Hur H, Min BS, et al. Predictive factors for lymph node metastasis in submucosal invasive colorectal carcinoma: a new proposal of depth of invasion for radical surgery. World J Surg 2018;42:2635-41. https://doi.org/10.1007/s00268-018-4482-4

22 Belderbos TDG, van Erning FN, de Hingh IHJT, et al. Long-term recurrence-free survival after standard endoscopic resection versus surgical resection of submucosal invasive colorectal cancer: a population-based study. Clin Gastroenterol Hepatol 2017;15:403-11.e1. https://doi.org/10.1016/j.cgh.2016.08.041

23 Chen T, Zhang YQ, Chen WF, et al. Efficacy and safety of additional surgery after non-curative endoscopic submucosal dissection for early colorectal cancer. BMC Gastroenterol 2017;17:134. https://doi.org/10.1186/s12876-017-0701-y

24 Ha RK, Han KS, Sohn DK, et al. Histopathologic risk factors for lymph node metastasis in patients with T1 colorectal cancer. Ann Surg Treat Res 2017;93:266-71. https://doi.org/10.4174/astr.2017.93.5.266

25 Pai RK, Chen Y, Jakubowski MA, et al. Colorectal carcinomas with submucosal invasion (pT1): analysis of histopathological and molecular factors predicting lymph node metastasis. Mod Pathol 2017;30:113-22. https://doi.org/10.1038/modpathol.2016.166

26 Kim B, Kim EH, Park SJ, et al. The risk of lymph node metastasis makes it unsafe to expand the conventional indications for endoscopic treatment of T1 colorectal cancer: A retrospective study of 428 patients. Medicine 2016;95:e4373. https://doi.org/10.1097/md.0000000000004373

27 Machado I, Valera-Alberni M, Martínez de Juan F, et al. Histological factors predicting loco-regional lymph node metastasis in early invasive colorectal adenocarcinoma pT1. Gastroenterol Hepatol 2016;39:1-8. https://doi.org/10.1016/j.gastre.2015.12.004

28 Macias-Garcia F, Celeiro-Muñoz C, Lesquereux-Martinez L, et al. A clinical model for predicting lymph node metastasis in submucosal invasive (T1) colorectal cancer. Int J Colorectal Dis 2015;30:761-8. https://doi.org/10.1007/s00384-015-2164-3

29 Caputo D, Caricato M, La Vaccara V, et al. T1 colorectal cancer: poor histological grading is predictive of lymph-node metastases. Int J Surg 2014;12:209-12. https://doi.org/10.1016/j.ijsu.2013.12.012

30 Nishida T, Egashira Y, Akutagawa H, et al. Predictors of lymph node metastasis in T1 colorectal carcinoma: an immunophenotypic analysis of 265 patients. Dis Colon Rectum 2014;57:905–15. https://doi.org/10.1097/dcr.0000000000000168

31 Yoshii S, Nojima M, Nosho K, et al. Factors associated with risk for colorectal cancer recurrence after endoscopic resection of T1 tumors. Clin Gastroenterol Hepatol 2014;12:292-302.e3. https://doi.org/10.1016/j.cgh.2013.08.008

32 Suh JP, Youk EG, Lee EJ, et al. Endoscopic submucosal dissection for nonpedunculated submucosal invasive colorectal cancer: is it feasible?. Eur J Gastroenterol Hepatol 2013;25:1051-9. https://doi.org/10.1097/meg.0b013e328361dd39

33 Wada H, Shiozawa M, Sugano N, et al. Lymphatic invasion identified with D2-40 immunostaining as a risk factor of nodal metastasis in T1 colorectal cancer. Int J Clin Oncol 2013;18:1025-31. https://doi.org/10.1007/s10147-012-0490-9

34 Kobayashi H, Mochizuki H, Morita T, et al. Characteristics of recurrence after curative resection for T1 colorectal cancer: Japanese multicenter study. J Gastroenterol 2011;46:203-11. https://doi.org/10.1007/s00535-010-0341-2

35 Ishii H, Ikegami M, Kobayashi H, et al. Risk factors for lymph node metastasis in submucosal invasive colon cancer: Emphasis on comparison between vessel permeation and budding. Tokyo Jikeikai Ika Daigaku Zasshi 2010;125:19-32.

36 Choi PW, Yu CS, Jang SJ, et al. Risk factors for lymph node metastasis in submucosal invasive colorectal cancer. World J Surg 2008;32:2089-94. https://doi.org/10.1007/s00268-008-9628-3

37 Kazama S, Watanabe T, Ajioka Y, et al. Tumour budding at the deepest invasive margin correlates with lymph node metastasis in submucosal colorectal cancer detected by anticytokeratin antibody CAM5.2. Br J Cancer 2006;94:293-8. https://doi.org/10.1038/sj.bjc.6602927

38 Wang H-S, Liang W-Y, Lin T-C, et al. Curative resection of T1 colorectal carcinoma: risk of lymph node metastasis and long-term prognosis. Dis Colon Rectum 2005;48:1182-92. https://doi.org/10.1007/s10350-004-0935-y

39 Tomoo W, Shinji K, Ken K, et al. Clinical significance of tumor surface desmoplastic reaction as a predictor of lymph node metastasis in colorectal cancer with submucosal invasion. Japanese Journal of Gastroenterological Surgery 2005;38:1675-83. https://doi.org/10.5833/jjgs.38.1675

40 Yamamoto S, Watanabe M, Hasegawa H, et al. The risk of lymph node metastasis in T1 colorectal carcinoma. Hepatogastroenterology 2004;51:998-1000. PMID: 15239233

41 Sakuragi M, Togashi K, Konishi F, et al. Predictive factors for lymph node metastasis in T1 stage colorectal carcinomas. Dis Colon Rectum 2003;46:1626-32. https://doi.org/10.1007/bf02660767

42 Tsuruta O, Tsuji Y, Kawano H, et al. Indication for endoscopic resection of submucosal colorectal carcinoma: special reference to lymph node metastasis. Diagn Ther Endosc 2000;6:101-9. https://doi.org/10.1155/dte.6.101

43 Coverlizza S, Risio M, Ferrari A, et al. Colorectal adenomas containing invasive carcinoma. Colorectal adenomas containing invasive carcinoma. Pathologic assessment of lymph node metastatic potential. Cancer 1989;64:1937-47. https://doi.org/10.1002/1097-0142(19891101)64:9<1937::aid-cncr2820640929>3.0.co;2-x
