# Supplementary material for: Diagnostic Accuracy of Highest-Grade or Predominant Histological Differentiation of T1 Colorectal Cancer in Predicting Lymph Node Metastasis: A Systematic Review and Meta-Analysis
Source: Clin Transl Gastroenterol. 2024 Jan 2;15(3):e00673. doi: 10.14309/ctg.0000000000000673 (PMC10962900; doi:10.14309/ctg.0000000000000673)
Supplement: Supplementary file 6 [file ct9-15-e00673-s006.docx]

**Supplemental Table 2**. Sensitivity analyses

| Sensitivity analysis | Differentiation classification | No. of studies | Sensitivity | 95% CI | Specificity | 95% CI |
| --- | --- | --- | --- | --- | --- | --- |
| Only surgical cases | Highest | 21 | 0.17 | 0.12–0.24 | 0.94 | 0.92–0.96 |
|  | Predominant | 12 | 0.05 | 0.03–0.09 | 0.99 | 0.97–0.99 |
| Only our criteria | Highest | 19 | 0.22 | 0.16–0.29 | 0.92 | 0.90–0.94 |
|  | Predominant | 12 | 0.05 | 0.03–0.08 | 0.99 | 0.98–0.99 |

CI confidence interval
